# ﻿Key to the Chinese species of the subgenus Sphodromimus Casale, 1984 (Carabidae, Chlaeniini, *Chlaenius*) with descriptions of two new species

**DOI:** 10.3897/zookeys.1135.93843

**Published:** 2022-12-12

**Authors:** Yuyao Qin, Christoph Germann, Hongbin Liang

**Affiliations:** 1 College of Life Sciences, Hebei University, Baoding 071002, China Hebei University Baoding China; 2 Key Laboratory of Zoological Systematics and Evolution, Institute of Zoology, Chinese Academy of Sciences, Beijing 100101, China Institute of Zoology, Chinese Academy of Sciences Beijing China; 3 Naturhistorisches Museum Basel, Augustinergasse 2, CH-4051 Basel, Switzerland Naturhistorisches Museum Basel Basel Switzerland

**Keywords:** Coleoptera, distribution, genitalia, ground beetles, taxonomy

## Abstract

The subgenus Sphodromimus Casale, 1984 in China has been studied, revealing two new species: Chlaenius (Sphodromimus) caperatus**sp. nov.** from Hunan Province and Chlaenius (Sphodromimus) yinggelingensis**sp. nov.** from Hainan Province. A new replacement name is proposed for C. (Sphodromimus) wrasei (Kirschenhofer, 2003) [nec Chlaenius (Lithochlaenius) wrasei Kirschenhofer, 1997]: Chlaenius (Sphodromimus) davidi**nom. nov.**. The status of Chlaenius (Sphodromimus) enleensis Mandl, 1992 is upgraded from subspecies to full species, and Chlaenius (Sphodromimus) tamdaoensis Kirschenhofer, 2003 is proposed as its new synonym. Chlaenius (Sphodromimus) pilosus (Casale, 1984) is reported as a new record from China. A key to all known species of the subgenus Sphodromimus from China is provided.

## ﻿Introduction

*Vachinius* Casale, 1984 was erected as a genus for *Pristonychussubglaber* Andrewes, 1937, and Sphodromimus Casale, 1984 was erected as a subgenus of Vachinius at the same time (Casale, 1984). Recently, both *Vachinius* and *Sphodromimus* were considered subgenera of the genus *Chlaenius* Bonelli, 1810 (Azadbakhsh and Kirschenhofer 2019). *Chlaeniuspeterseni* (Louwerens, 1967), *C.flavofemoratus* Laporte, 1834, and *C.tamdaoensis* Kirschenhofer, 2003 were transferred from the subgenus Haplochlaenius to the subgenus Sphodromimus by Azadbakhsh and Kirschenhofer (2019). To date, in total 14 species are recognized in the subgenus Sphodromimus, distributed in the Oriental Region, e.g., China, Indonesia, Laos, Myanmar, Nepal, Philippines, Thailand, Vietnam (Morvan 1991, 1997; Lassalle 2001; Kirschenhofer 2003, 2012; Brunk 2015; Zettel 2020).

The subgenus differs from other subgenera of genus *Chlaenius* mainly by its large size (length 19.0–26.0 mm), elytral intervals densely punctate and pubescent, slightly convex, not costulate, and the apical lamella of the aedeagus is denticulate on the dorsal side (Casale 1984). Before the present study, four species of this subgenus had been recorded from China: Chlaenius (Sphodromimus) deuvei (Morvan, 1997), Chlaenius (Sphodromimus) flavofemoratus Laporte, 1834, Chlaenius (Sphodromimus) hunanus (Morvan, 1997), and Chlaenius (Sphodromimus) wrasei (Kirschenhofer, 2003). When examining specimens from south China, we found two new species and a new country record based on comparison with types and/or original descriptions. In this paper, we describe the new species and report the newly found one, upgrade one subspecies to full species, propose a new replacement name, and provide a revised key to all known species of subgenus Sphodromimus in China.

## ﻿Materials and methods

Specimens examined during our study are deposited in the following collections:

**CAS**California Academy of Science, San Francisco, USA;

**DWC** working collection David W. Wrase, Gusow-Platkow, Germany (part of Zoologische Staatssammlung, München);

**IZAS**Institute of Zoology, Chinese Academy of Sciences, Beijing, China;

**MNHN**Muséum National d’Histoire Naturelle, Paris, France;

**NHMB**Naturhistorisches Museum Basel, Switzerland;

**SCAU** South China Agriculture University, Guangzhou, China.

Abbreviations for measurements used in the paper are as follows:

**BL** length of body, measured from the apical margin of the labrum to the elytral apex;

**BW** width of body, measured across the elytral greatest width;

**EL** length of elytra, measured from the base of the scutellum to the elytra apex;

**ML** length of metepisternum, measured along its outer side;

**MW** width of metepisternum, measured along its anterior side;

**PAW** width of apical pronotum, measured between the apices of the anterior angle;

**PBW** width of basal pronotum, measured along its basal margin;

**PL** length of pronotum, measured along its median line;

**PW** width of pronotum, measured across its greatest width.

The methods of dissection, illustrations, and measurements mainly follow our previous work (Shi et al. 2013a, b). Terminology of female genitalia follows Deuve (1993) and Liebherr and Will (1998).

## ﻿Taxonomic account

### ﻿Subgenus Sphodromimus

Taxon classificationAnimaliaColeopteraCarabidae

Casale, 1984

C1F49D6A-1F58-5AFB-9C71-D7D2124DAB99

Sphodromimus Casale, 1984: 372; Morvan 1991: 60 (described new species); Morvan 1997: 16 (described new species); Lorenz 1998: 320 (catalogue); Lassalle 2001: 240 (described new species); Kirschenhofer 2003: 32 (described new species); Lorenz 2005: 341 (catalogue); Kirschenhofer 2012: 84 (described new species); Kirschenhofer 2013: 9 (new combination from Haplochlaenius); Brunk 2015: 5 (described new species); Kirschenhofer 2017: 497 (catalogue); Azadbakhsh and Kirschenhofer 2019: 1 (Sphodromimus considered subgenus of Chlaenius); Zettel 2020: 29 (described new species).

#### Type species.

*Vachiniusholzschuhi* (Casale, 1984) (type locality: East Nepal, Tashigaon 2100 m; holotype in NHMB), by original designation.

#### Diagnosis.

*Sphodromimus* can be distinguished from other subgenera in *Chlaenius* by the following character combinations: body large, BL 19–26 mm; body black or metallic colored, luster matt or strongly shiny, antennae, mandibles usually dark brown, elytra black, ventral side black; head finely punctate; penultimate labial palpomere with 5–7 setae, apex truncate; antennae long, antennomere 3 longer than 4; mentum tooth stout, bifid; pronotum long, with sides usually sinuate before posterior angles, posterior lateral seta situated before posterior angles, anterior lateral seta absent; proepisterna sparsely punctate and pubescent; elytral intervals flat or convex, not ribbed, densely punctate and pubescent, basal margin reaching the scutellum; hind wings reduced in all species except *Chlaeniusflavofemoratus* Laporte, 1834 and *Chlaeniuspeterseni* (Louwerens, 1967); prosternal process unbordered at apex; metepisterna wider than long in all species except *C.flavofemoratus* and *C.peterseni*, coarsely punctate, pubescent; legs sparsely pubescent, tarsi nearly smooth dorsally, claws simple, protibiae sulcate on dorsal side; abdominal sternites finely punctate laterally; apical lamella of aedeagus denticulate on dorsal side; apical gonocoxite without ensiform setae; receptaculum very short to absent.

#### Comparisons.

This subgenus is most similar to subgenera *Haplochlaenius* Lutshnik and *Vachinius* Casale, but differs in having elytra with intervals flat or slightly convex, densely punctate and pubescent, and with basal margin complete, connected with scutellum (intervals strongly ribbed in *Haplochlaenius*, basal margin obsolete near scutellum; intervals smooth in *Vachinius*; cfr. Azadbakhsh and Kirschenhofer 2019).

#### Species and distribution.

Subgenus is composed of 16 species distributed in the Oriental Region (China, Indonesia, Laos, Myanmar, Nepal, Philippines, Thailand, Vietnam), including the two new species described below.

### ﻿Key to species of subgenus Sphodromimus Casale, 1984 from China

**Table:** 

1	Pronotum copper-green to violet, shining, with metallic luster	**2**
–	Pronotum black and matt	**4**
2	Pronotum cordate, widest at apical third, anterior angles projected forward, strongly sinuate before posterior angles (Figs 21, 23); hind wings reduced; metepisterna short, MW/ML = 1.03–1.17 (Figs 12, 15)	**3**
–	Pronotum subquadrate, widest at middle, anterior angles not projected forward, lateral margins rounded or straight before posterior angles (Fig. 22); hind wings developed; metepisterna long, MW/ML = 0.75–0.92 (Fig. 14)	***C.flavofemoratus* Laporte, 1834**
3	Legs entirely black (Fig. 9A, B). Guangdong, Xinyi; Guangxi, Daming Shan	***C.davidi* nom. nov.**
–	Distal half of femora red-brown (Fig. 4A–D). Hainan, Yinggeling	***C.yinggelingensis* sp. nov.**
4	Posterior angles of pronotum slightly projected backward (Fig. 19); apex of apical lamella of aedeagus concaved in the middle, both sides thickened, each with a denticulation (Fig. 27A); receptaculum tiny, seminal canal short (Fig. 36A). Guangxi, Mao’er Shan; Guangxi, Huaping	***C.deuvei* (Morvan, 1997)**
–	Posterior angles of pronotum not projected (Figs 17, 18, 20); apex of apical lamella of aedeagus truncated or rounded	**5**
5	Pronotum subquadrate, nearly as long as wide (PW/PL = 1.02–1.07), lateral margins straight before posterior angles (Fig. 20); apex of apical lamella of aedeagus truncated; right side of median lobe with a large denticulation, left side with a small denticulation (Fig. 28A). Yunnan, Dawei Shan; Vietnam, the Black River	***C.pilosus* (Casale, 1984)**
–	Pronotum cordate, much wider than long (PW/PL = 1.18–1.26), lateral margins faintly sinuate before posterior angles (Fig. 17, 18); apex of apical lamella of aedeagus untruncated	**6**
6	Pronotum with apical width equal to or slightly shorter than basal width (PAW/PBW = 0.99–1.00) (Fig. 17); apex of apical lamella of aedeagus rounded (Fig. 25A); receptaculum long, seminal canal short (Fig. 34A). Hunan, Guidong	***C.caperatus* sp. nov.**
–	Pronotum apical width clearly shorter than basal width of pronotum (PAW/PBW = 0.87–0.97) (Fig. 18); apical lamella of aedeagus triangular (Fig. 26A); receptaculum short, seminal canal long (Fig. 35A). Hunan Jiuyi Shan; Guangdong, Nanling	***C.hunanus* (Morvan, 1997)**

### ﻿ Chlaenius (Sphodromimus) caperatus sp. nov.

Taxon classificationAnimaliaColeopteraCarabidae

DC833AEC-C9B2-5AB5-B79F-C1F231D614DF

https://zoobank.org/DC3445D1-970D-43DE-B622-8EA561F22632

**Figure 1.**
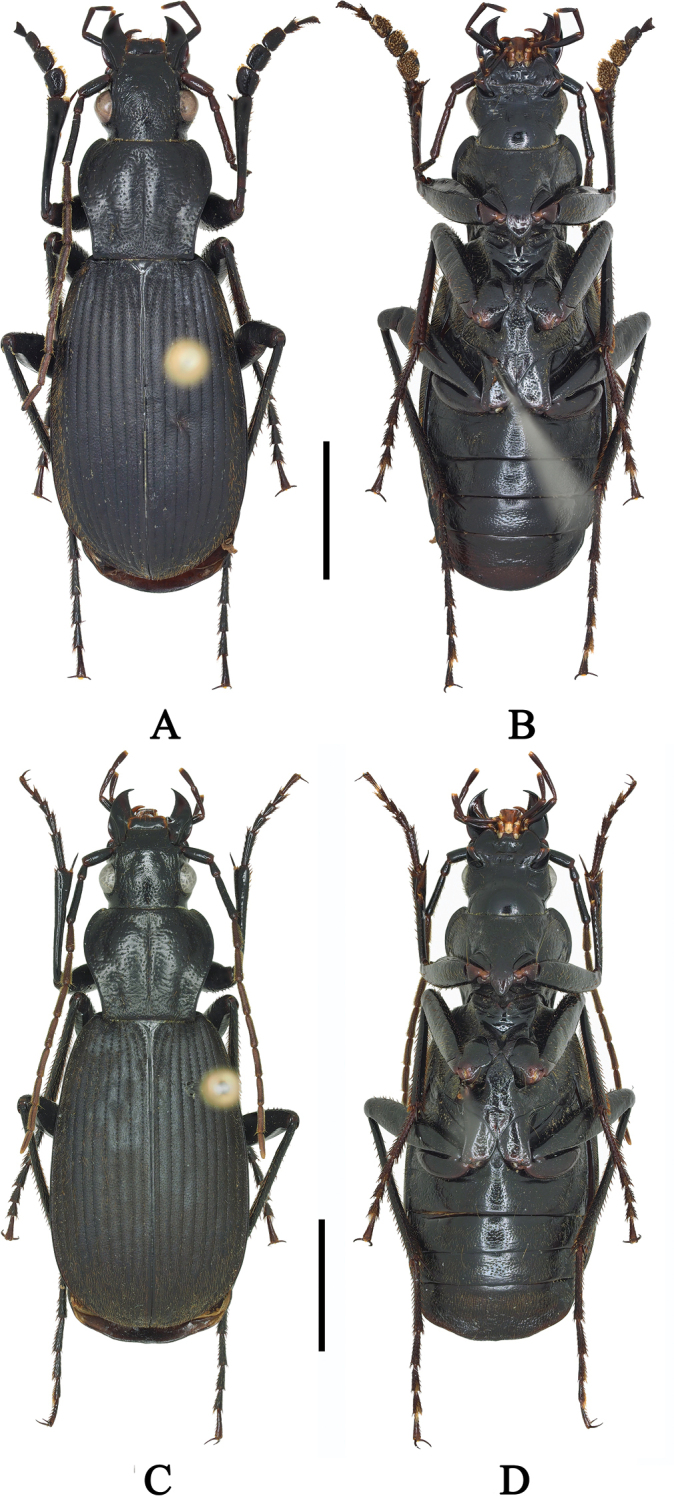
**A, B**Chlaenius (Sphodromimus) caperatus sp. nov. (holotype, Hunan, Guidong) **C, D**C. (Sphodromimus) caperatus sp. nov. (female paratype, Hunan, Guidong). Scale bars: 5.0 mm.

Figs 1A–D , 11 , 17 , 25A–E , 34A–C , 40

#### Type locality.

China, Hunan, Guidong: Qiyun Shan (25.9010°N, 114.0068°E), altitude 1299.12 m.

#### Type material.

***Holotype*.** Male (IZAS) [genitalia dissected and glued on plastic film pinned under specimen], Hunan, Guidong, Qiyun Shan, 25.9010°N, 114.0068°E, 1299.12 m, 2017.XI.12–14, S.P. Yu, Y.Z. Liu leg., Institute of Zoology, IZAS/Holotype Chlaenius (Sphodromimus) caperatus sp. nov. des. by Y.Y. Qin, 2022 [red label].

***Paratypes*.** Total 8 specimens: 2 ♂♂ and 3 ♀♀ (IZAS), same data as holotype; 1 ♀ (IZAS), Hunan, Guidong, Qiyun Shan, 25.9007°N, 114.01318°E, 1487.17 m, 2017.XI.12–14, S.P. Yu, Y.Z. Liu leg., Institute of Zoology, IZAS; 1 ♂ and 1 ♀ (SCAU) “Hunan, Guidong, Dongluo, Chishuixian, 1350–1450 m, 2011.XII.1, M.Y. Tian, Q. Gao, F.F. Sun leg., SCAU. All paratypes also bear the following label: Paratype. Chlaenius (Sphodromimus) caperatus sp. nov. des. by Y.Y. Qin, 2022 [red label].

#### Diagnosis.

Dorsum black. PW/PL = 1.18–1.21; PAW/PBW = 0.99–1.00 (Fig. 17); pronotum with anterior angles rounded, moderately projected forward; disc sparsely punctate and pubescent with deep transverse rugosities, but with a glabrous area in the middle. Elytral intervals flat, densely punctate and pubescent. Hind wings reduced. Metepisterna short, MW/ML = 1.1–1.3 (Fig. 11). Apex of apical lamella rounded (Fig. 25A–E).

#### Comparisons.

**Figures 2, 3.**
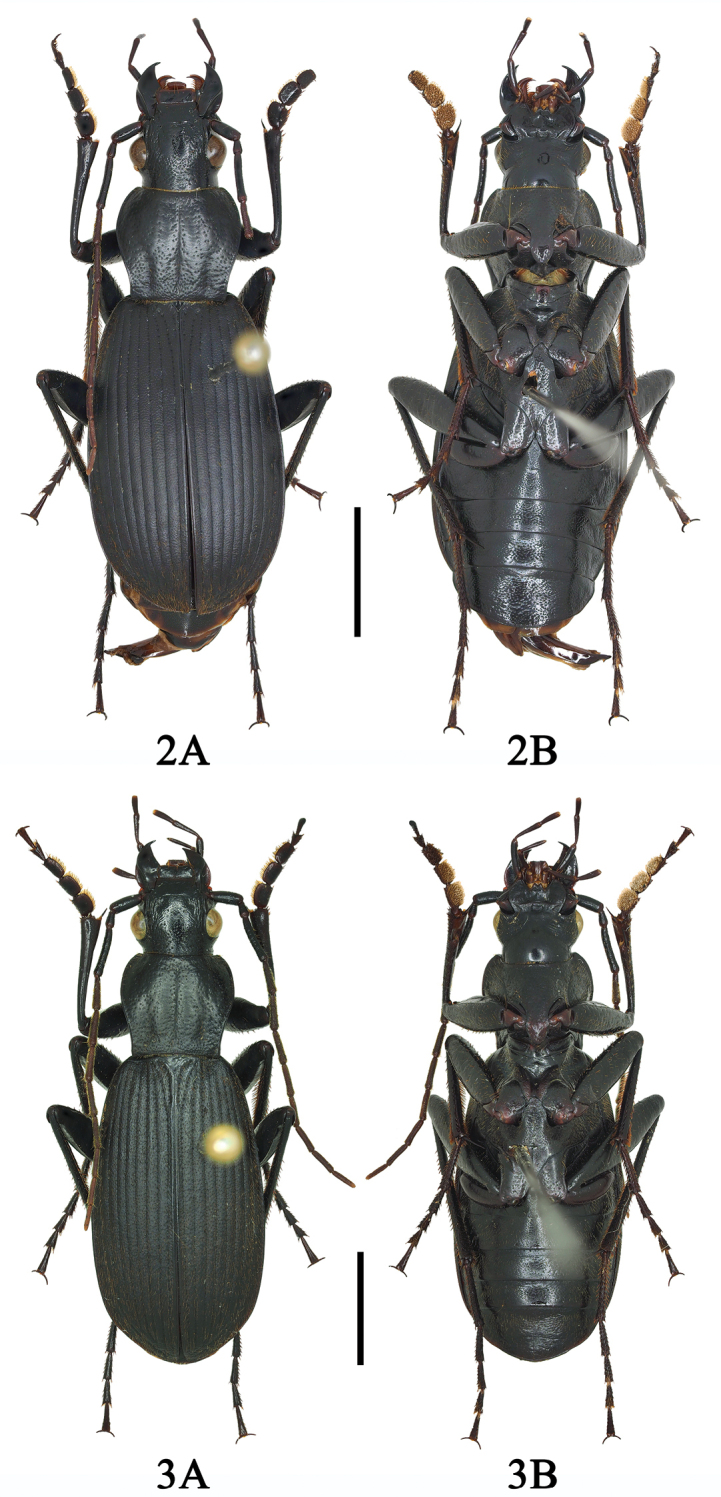
**2A, B**Chlaenius (Sphodromimus) hunanus (Morvan, 1997) (male, Guangdong, Nanling) **3A, B**C. (Sphodromimus) deuvei (Morvan, 1997) (male, Guangxi, Huaping). Scale bars: 5.0 mm.

This new species is most similar to Chlaenius (Sphodromimus) hunanus (Morvan, 1997) (Fig. 2A, B), sharing the large size, shape of pronotum, black elytra, and reduced hind wings, but can be distinguished from the latter by: (1) PAW/PBW = 0.99–1.00 (Fig. 17), (0.87–0.97 in *C.hunanus*, Fig. 18); (2) apex of lamella of median lobe rounded (apex of lamella triangular in *C.hunanus*, Fig. 26A–E); (3) in female genitalia, receptaculum longer (shorter in *C.hunanus*, Fig. 35A–C), and seminal canal shorter (longer in *C.hunanus*, Fig. 35A–C).

#### Description.

BL = 20.3–22.0 mm, BW = 7.6–8.9 mm [BL = 21.0 mm, BW = 7.0 mm in holotype], PAW = 3.6–3.9 mm, PBW = 3.6–3.9 mm, PW = 5.0–5.5 mm, PL = 4.3–4.5 mm, MW = 1.9–2.0 mm, ML = 1.5–1.8 mm. Head, pronotum, elytra, legs, and venter black; antennae, labial and maxillary palpi, apex of mouthparts, and tarsomeres dark brown.

***Head*.** Vertex punctate and pubescent with a glabrous and rugose area in the middle; antennae long, reaching middle of elytra; antennomere 3 ~ 1.5× longer than antennomere 4.

***Pronotum*** cordiform, PW/PL = 1.18–1.21 (Fig. 17), widest at apical third; anterior margin slightly concave, its width equal to its basal width, PAW/PBW = 0.99–1.00; lateral margins rounded before middle, then distinctly narrowed to base, faintly sinuate before posterior angles; anterior angles rounded, moderately projected forward; posterior angles obtuse, slightly sharp at tips; disc gently convex, sparsely punctate and pubescent, with deep transverse rugosities, with a small glabrous area in the middle; median line distinct, not reaching anterior margin and base; basal foveae deep and long, punctate, pubescent.

***Elytra*** elongate, EL/BW = 1.45–1.61; gently convex, widest near posterior third, rounded at apex in males, subtruncate in females; striae with fine punctures; parascutellar striae well developed; parascutellar pores present; intervals flat, densely punctate and pubescent; sutural angles sharp; hind wings reduced.

***Venter*** densely punctate, pubescent, metepisterna (Fig. 11) short, MW/ML = 1.25–1.33; abdominal sternites III–VI with one setiferous puncture each side, sternite VII with one pair of setiferous punctures in males, two pairs in females; all abdominal sternites with distinct impressions laterally.

***Legs*** long and slender; tarsi nearly smooth dorsally.

***Male genitalia*.** Median lobe (Fig. 25B–E) long, strongly bent to ventral side; apical orifice opened dorsally, long and wide, not reaching basal bulb, slightly turned to left side; in dorsal view, apical lamella (Fig. 25A) slightly bent to left side, length nearly equal to its basal width, apex rounded; in left lateral view, apical portion distinctly bent into a hook ventrally (Fig. 25B, E), basal orifice ~ 90 ° relative to preapical shaft; left paramere large and round; right paramere helical carved; endophallus with coiled flagellum; basal part of flagellum strongly bent with an oval sclerite facing the right; apical part of flagellum distinctly bent with a triangular bursa.

***Female genitalia*.** Spermatheca (Fig. 34A–C) with seminal canal ~ 15× as long as receptaculum; receptaculum short linear; seminal canal inserted at base of common oviduct; spermathecal gland rounded and inserted near apex of seminal canal; villous canal long, tortuously contorted, adhered to common oviduct.

#### Distribution.

(Fig. 40) This species is known only from Guidong, Hunan. Its distribution seems isolated by a mountain barrier from *C.hunanus*.

#### Etymology.

The new species *caperatus* is named for its rugosity on the vertex, pronotal disc, and abdominal sternites.

#### Remarks.

We have not examined the types of C. (S.) hunanus, but Dr. Deuve (MNHN) kindly helped us to compare types and sent us illustrations of the apical lamella of holotype. More than 60 specimens collected from Nanling, Guangdong fit Morvan’s descriptions and illustration (1997: 17, fig. 13), and we determined them as C. (S.) hunanus. Nanling is located ~ 100 km north of the type locality of C. (S.) hunanus Jiuyi Shan, Hunan (Fig. 40).

### ﻿ Chlaenius (Sphodromimus) yinggelingensis sp. nov.

Taxon classificationAnimaliaColeopteraCarabidae

3A0D0D88-4CFB-57FF-8462-0CC29715BF18

https://zoobank.org/95948EBD-4279-457D-BB06-0A501D8198D9

**Figure 4.**
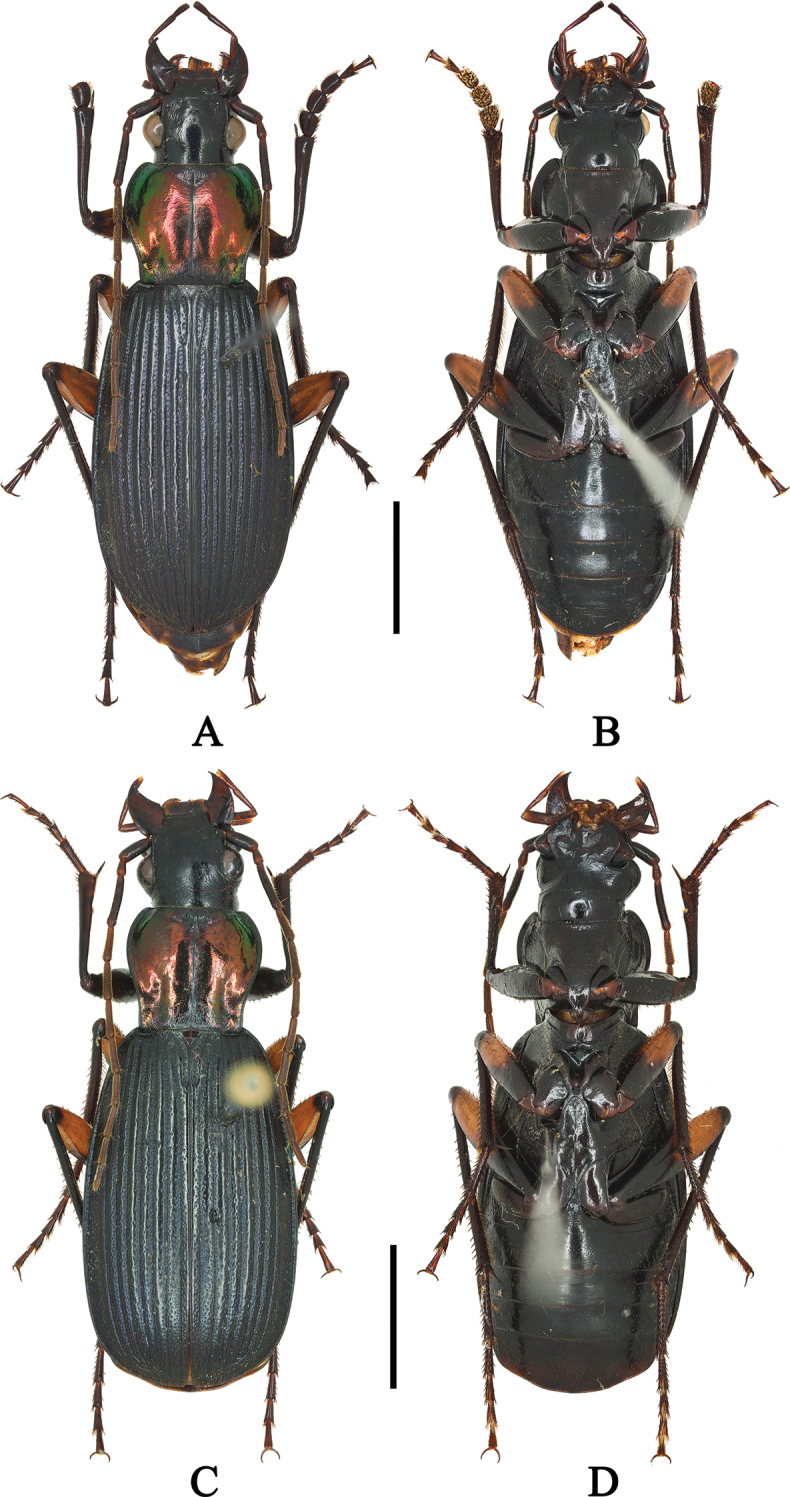
**A, B**Chlaenius (Sphodromimus) yinggelingensis sp. nov. (holotype, Hainan, Yinggeling) **C, D**C. (S.) yinggelingensis sp. nov. (female paratype, Hainan, Jianfengling). Scale bars: 5.0 mm.

Figs 4A–D , 12 , 21 , 29A–E , 37A–C , 40

#### Type locality.

China, Hainan, Yinggeling.

#### Type material.

***Holotype*.** Male (IZAS) [genitalia dissected and glued on plastic film pinned under specimen], Hainan, Yinggeling, 2009.V.11, Xinlei Huang leg., Institute of Zoology, IZAS/Holotype Chlaenius (Sphodromimus) yinggelingensis sp. nov. des. by Y.Y. Qin, 2022 [red label].

***Paratypes*.** Total 4 specimens: 1 ♂ (IZAS), same data as holotype; 2 ♀♀ (IZAS), Hainan, Jianfengling, Mingfenggu, 947 m, 2015.I.23, Deyao Zhou leg., Institute of Zoology, IZAS; 1♀ (IZAS), Hainan, Wuzhishan, 18°54'N, 109°41'E, 1000–1600 m, 2012.IV.18, PAN & LI leg. All paratypes also bear the following label: Paratype. Chlaenius (Sphodromimus) yinggelingensis sp. nov. des. by Y.Y. Qin, 2022 [red label].

#### Diagnosis.

Pronotum metallic coppery to green. PW/PL = 1.12–1.21; PAW/PBW = 0.84–0.91 (Fig. 21); pronotum cordate with posterior angles right angled, rounded at tips; disc sparsely punctate and pubescent with shallow, transverse rugosities. Elytral intervals distinctly convex, with a row of setae laterally and sparse setae centrally. Hind wings reduced. Metepisterna short or width nearly equal to length; MW/ML = 1.03–1.17 (Fig. 12). Distal half of femora red-brown, the rest of legs black.

#### Comparisons.

This new species is similar to Chlaenius (Sphodromimus) flavofemoratus (Figs 7A–C), in having a large size, coloration of the pronotum and femora, and the absence of a spermatheca, but can be distinguished from the latter by: (1) pronotum cordate (subquadrate in *C.flavofemoratus*); (2) metepisterna wider than or nearly equal to long (longer than wide in *C.flavofemoratus* as in Fig. 14); (3) hind wings reduced (developed in *C.flavofemoratus*); (4) interval convex throughout (convex basally, flat apically in *C.flavofemoratus*).

**Figures 5, 6.**
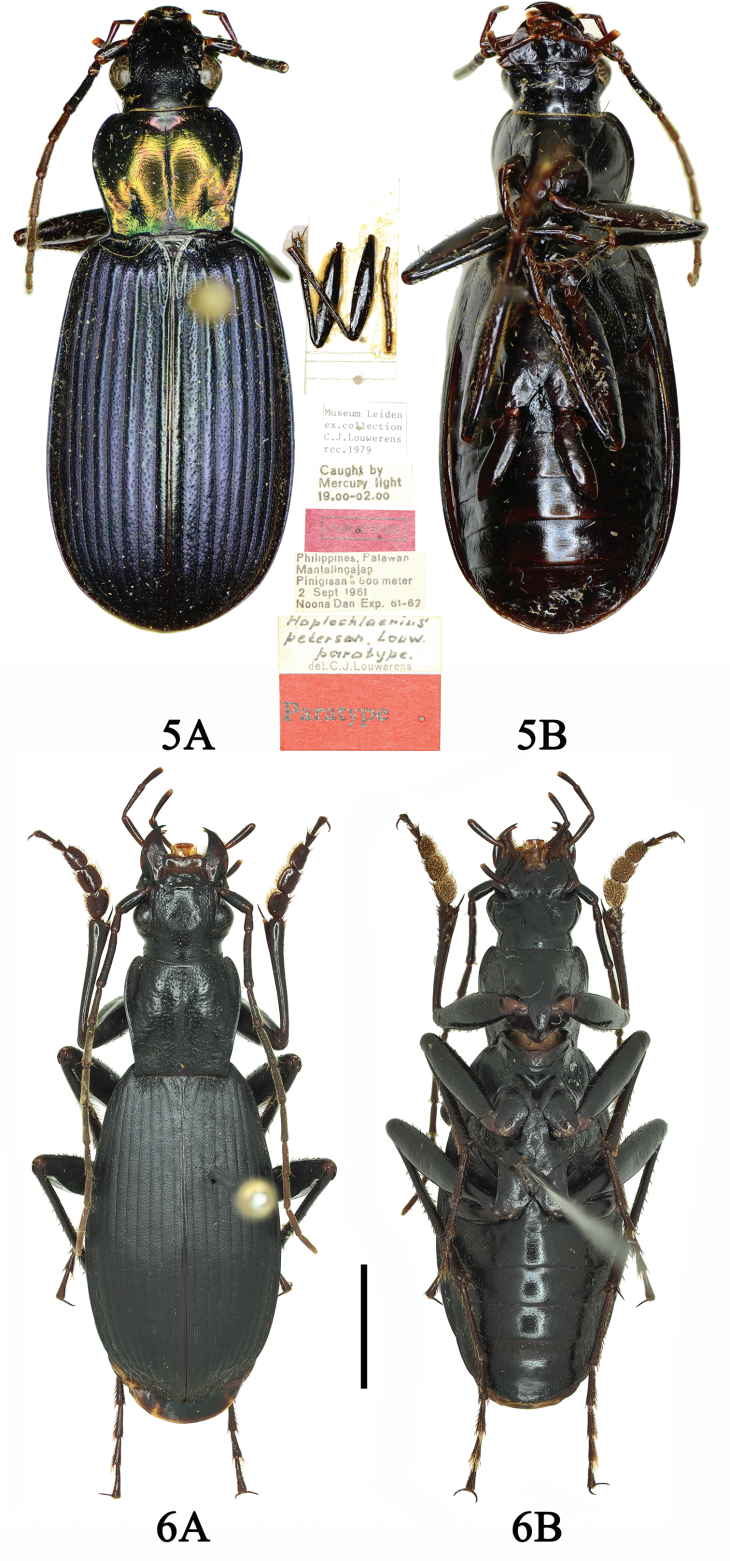
**5A, B**Chlaenius (Sphodromimus) peterseni (Louwerens, 1967) (female paratype, Philippine, photos by Shi Hongliang) **6A, B**C. (Sphodromimus) pilosus (Casale, 1984) (male, Yunnan, Pingbian). Scale bar: 5.0 mm.

It is also similar to Chlaenius (Sphodromimus) peterseni (Louwerens, 1967) from the Philippines in having pronotum with green metallic luster and elytral intervals slightly convex, but differs in having meso- and metafemora with yellow coloration in the middle, pronotum with lateral margins sinuate before posterior angles, hind wings reduced (femoral black, pronotum with lateral margins straight, and hind wings developed in *C.peterseni*, Fig. 5A, B)

#### Description.

BL = 20.9–21.6 mm, BW = 7.5–7.9 mm [BL = 21.3 mm, BW = 7.7 mm in holotype], PAW = 3.4–3.6 mm, PBW = 3.7–4.0 mm, PW = 4.8–5.3 mm, PL = 4.1–4.5 mm, MW = 1.7–1.8 mm, ML = 1.5–1.6 mm. Head, elytra, and venter black; pronotum metallic green to metallic coppery; antennae, labial and maxillary palpi, apex of mouthparts, and tarsomeres dark brown; distal half of femora red-brown, the rest of legs black.

***Head*.** Vertex sparsely, finely punctate and pubescent; antennae long, reaching middle of elytra; antennomere 3 ~ 1.5× longer than antennomere 4.

***Pronotum*** cordiform, PW/PL = 1.12–1.21 (Fig. 21), widest at apical third; anterior margin slightly concave, PAW/PBW = 0.84–0.91; lateral margins rounded before middle, then distinctly narrowed to base, straight before posterior angles; anterior angles rounded, moderately projected forward; posterior angles almost right angled, slightly sharp at tips; disc sparsely punctate and pubescent, with shallow transverse rugosities, without glabrous area in the middle; median line distinct, not reaching anterior margin and base; basal foveae deep and long, punctate, pubescent.

***Elytra*** elongate, EL/BW = 1.67–1.73, gently convex, widest near posterior third, rounded at apex in males, subtruncate in females; basal margin sinuate, reaching the scutellum (but slightly obsolete on one side in a female); striae with deep punctures; parascutellar striae well developed; parascutellar pores present; intervals distinctly convex, with a row of setae laterally and sparse setae centrally; sutural angles obtuse; hind wings reduced.

***Venter*** sparsely pubescent, punctate; metepisterna (Fig. 12) short or width nearly equal to length, MW/ML = 1.03–1.17; abdominal sternites III–VI with one setiferous puncture each side, sternite VII with one pair of setiferous punctures in males, two pairs in females; all abdominal sternites with a few impressions laterally.

***Legs*** long and slender; tarsi nearly smooth dorsally.

***Male genitalia*.** Median lobe (Fig. 29B–E) long, strongly bent to ventral side; apical orifice opened dorsally, long and wide, not reaching basal bulb; in dorsal view, apical lamella (Fig. 29A) wide and short, wider than long, apex subtruncate, each side distinctly widened and thickened; in left lateral view, apex with a denticulation bent to back; in right lateral view, each side convex into a denticulation, right side smaller than left side, basal orifice ~ 90° relative to preapical shaft; left paramere large and oval; right paramere helically curved; endophallus with flagellum thick and straight; basal part of flagellum with a disciform sclerite facing the left.

***Female genitalia*.** Bursa copulatrix (Fig. 37A–C) asymmetric, base with a bifid irregular protrusion; villous canal long, tortuously contorted, adhered to common oviduct; spermatheca and spermathecal gland absent.

#### Distribution.

(Fig. 40) China (Hainan).

#### Etymology.

The new species *yinggelingensis* is named for the type locality Yinggeling, Hainan.

#### Remarks.

We dissected two females in *C.yinggelingensis*, four in *C.davidi* and nine in *C.flavofemoratus*. As a result, we could not find either spermatheca or spermathecal gland. The absence of spermatheca is uncommon in Carabidae and only occasionally found in Trechini (Deuve 1993: fig. 250). They are also absent at least in other two species of the subgenus, C. (Sphodromimus) davidi and C. (Sphodromimus) flavofemoratus (see female genitalia descriptions below).

### ﻿ Chlaenius (Sphodromimus) pilosus

Taxon classificationAnimaliaColeopteraCarabidae

(Casale, 1984), new record from China

148A3BC9-8D23-5200-AD3F-0D3C105A4752

Figs 6A, B , 13 , 20 , 28A–E , 40

Vachinius (Sphodromimus) pilosus Casale, 1984: 379; Lorenz 1998: 320 (catalogue); Lorenz 2005: 341 (catalogue); Brunk and Kirschenhofer 2016: 54 (record); Azadbakhsh and Kirschenhofer 2019: 1 (transferred from genus Vachinius).

#### Type locality.

Vietnam, Chapa, Tonkin, Coll. J. Clermont.

#### Material examined.

China – Yunnan Prov.: 4 ♂♂ (IZAS), Pingbian, Dawei Shan, 2100 m, 2010.V.23, X.D. Yang, X.Y. Zhu leg.

#### Diagnosis.

Dorsum black. PW/PL = 1.02–1.07; PAW/PBW = 0.98–1.00 (Fig. 20); pronotum subquadrate with anterior angles rounded, slightly projected forward; disc densely and completely rugose, punctate. Elytral intervals flat and densely punctate and pubescent. Hind wings reduced. Metepisterna short, MW/ML = 1.3–1.4 (Fig. 13). Apical lamella of median lobe truncated, right side with a large denticulation, left side with a small denticulation (Fig. 28A–E), sometimes, such denticulation absent.

**Figures 7, 8.**
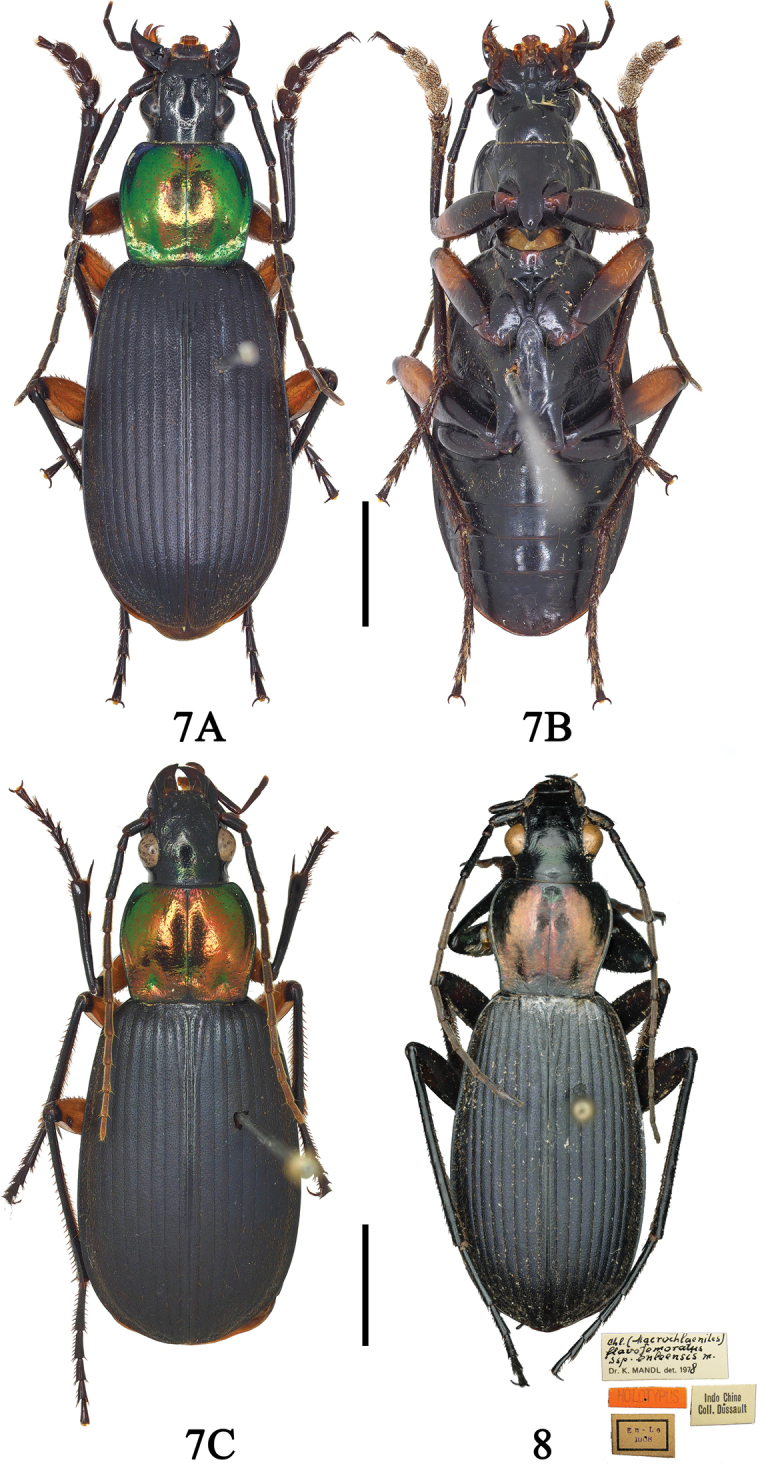
**7A, B**Chlaenius (Sphodromimus) flavofemoratus Laporte, 1834 (male, Yunnan, Menglun) **C**C. (Sphodromimus) flavofemoratus Laporte, 1834 (female, Yunnan, Menglun) **8**C. (Sphodromimus) enleensis Mandl, 1992 (holotype, Indo Chine) Scale bars: 5.0 mm.

#### Description.

BL = 19.6–21.9 mm, BW = 7.3–7.6 mm, PAW = 3.33–3.55 mm, PBW = 3.40–3.55 mm, PW = 4.35–4.55 mm, PL = 4.05–4.45 mm, MW = 1.65–1.80 mm, ML = 1.20–1.35 mm. Head, pronotum, elytra, legs, and venter black; antennae, labial and maxillary palpi, apex of mouthparts and tarsomeres dark brown.

***Head*.** Vertex punctate and pubescent with a rugose area; antennae long, reaching middle of elytra; antennomere 3 ~ 1.5× longer than antennomere 4.

***Pronotum*** subquadrate, PW/PL = 1.02–1.07 (Fig. 20), widest at apical third; anterior margin slightly concave, its width equal to width of basal margin, PAW/PBW = 0.98–1.00; lateral margins slightly bent before middle, then gently narrowed to base, straight before posterior angles; anterior angles rounded, slightly projected forward; posterior angles right angled, slightly rounded at tips; disc gently convex, sparsely punctate, pubescent, with a few transverse rugosities, without glabrous area in the middle; median line distinct, deep, reaching anterior margin and base; basal foveae deep, short, broad, punctate, and pubescent.

***Elytra*** elongate, EL/BW = 1.64–1.77; gently convex near anterior third, widest near posterior third, rounded at apex in males; striae with deep punctures; parascutellar striae well developed; parascutellar pores present; intervals flat, densely punctate and pubescent; sutural angles sharp; hind wings reduced.

***Venter*** densely punctate, pubescent, metepisterna (Fig. 13) short, MW/ML = 1.30–1.38; abdominal sternites III–VI with one setiferous puncture each side, sternite VII with one pair of setiferous punctures in males, two pairs in females; all abdominal sternites with distinct impressions laterally.

***Legs*** long and slender; tarsi nearly smooth dorsally.

***Male genitalia*.** Median lobe (Fig. 28B–E) stout, strongly bent to ventral side; apical orifice opening dorsally, long and wide, not reaching basal bulb; in dorsal view, apical lamella (Fig. 28A) wide and short, wider than long, apex truncated; in left lateral view, apical right side with a large denticulation towards the base and outside, basal left side only thickened and convex or convex into a small denticulation, basal orifice ~ 90 ° relative to preapical shaft; left paramere large and round; right paramere helically curved; endophallus with flagellum coiled; basal part of flagellum with a disciform sclerite facing the right; apical part of flagellum with strip of sclerite.

***Female genitalia*** unknown.

#### Distribution.

China (Yunnan), Vietnam.

#### Remarks.

The type locality is situated in the mountains of the Black River, northern Vietnam, not far from the Chinese frontier. Our identification is based on the original description and illustration of the male genitalia of the holotype by Casale (1984: 379, figs 11, 13).

### ﻿ Chlaenius (Sphodromimus) davidi nom. nov.

Taxon classificationAnimaliaColeopteraCarabidae

B5DB7516-49F2-5FBF-B9B9-635FA0BEBE81

**Figures 9, 10.**
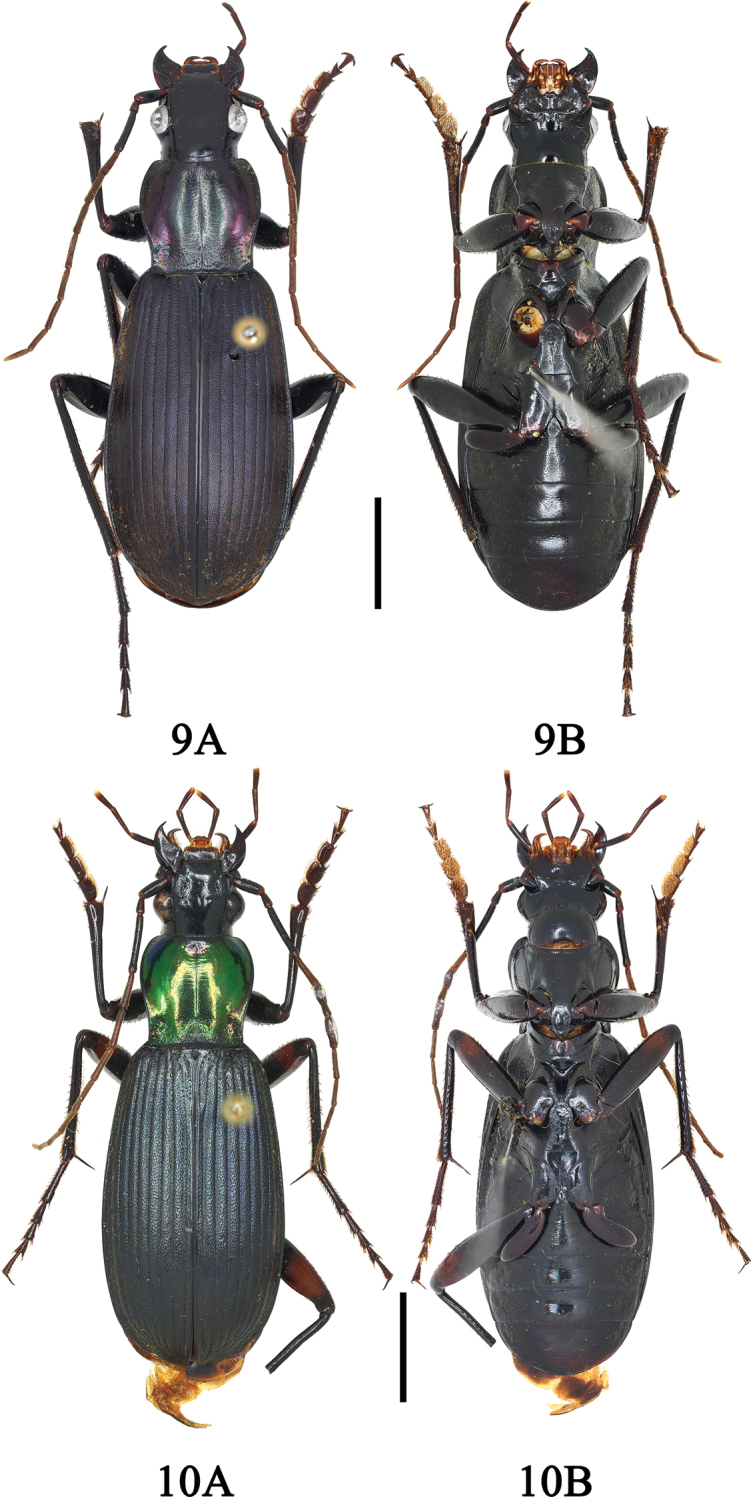
**9A, B**Chlaenius (Sphodromimus) davidi nom. nov. (male, Guangdong, Xinyi) **10A, B**C. (Sphodromimus) enleensis (male, Vietnam, Tam Dao). Scale bars: 5.0 mm.

Figs 9A, B , 15 , 23 , 31A–E , 39A–C , 40

Vachinius (Sphodromimus) wrasei Kirschenhofer, 2003: 37 (type locality: China, Guangdong); Lorenz 2005: 342 (catalogue); Brunk and Kirschenhofer 2016: 54 (record); Azadbakhsh and Kirschenhofer 2019: 1 (genus Chlaenius, subgenus Sphodromimus) [nec C. (Lithochlaenius) wrasei (Kirschenhofer, 1997)]

#### Type locality.

Guangdong, Xinyi, Datianding.

#### Material examined.

Total 10 specimens. China – **Guangdong**: Holotype female (DWC, photo), China, Guangdong, 1500 m, Xinyi: Datianding (22.16/111.15) -VIII-1997 – leg. Li/Holotypus, Chlaenius (Sphodromimus) wrasei sp. nov. det. Kirschenhofer, 2001[red label]/COLL WRASE, BERLIN; 1 ♂ and 4 ♀♀ (IZAS), Guangdong, Xinyi, Yunkai Shan, 1508.21m/22.291317°N, 111.209888°E, 2017.V.30, Y.Z. Liu, S.P. Yu leg., Inst. of Zoology; 2 ♀♀ (IZAS), Guangdong, Xinyi, Yunkai Shan, 1250.55 m/22.292692°N, 111.203833°E, 2017.V.31, Y.Z. Liu, S.P. Yu leg., Inst. of Zoology; **Guangxi**: 2 ♂♂ (IZAS), Guangxi Prov, Daming Shan, Tianping Protect Station, N 23.49811, E 108.43715/1230 m, 2011.V.27 N, Xinlei Huang Coll., Inst. of Zoology.

#### Diagnosis.

Pronotum fully metallic purple, or greenish purple. PW/PL = 1.09–1.11; PAW/PBW = 0.83–0.93 (Fig. 23); pronotum cordate with posterior angles nearly right angled, rounded at tips; disc sparsely punctate and pubescent, with dense, shallow, transverse rugosities, without glabrous area in the middle. Elytral intervals convex basally, flat apically, densely punctate and pubescent. Hind wings reduced. Metepisterna short, MW/ML = 1.11–1.15 (Fig. 15). Legs totally black.

#### Comparisons.

This species is most similar to Chlaenius (Sphodromimus) enleensis Mandl, 1992 (Figs 8, 10A, B), sharing the large size, general shape of apical lamella, metepisterna short (Figs 15, 16), and reduced hind wings, but can be distinguished from the latter by: (1) pronotum purple or greenish purple (Fig. 23) (green to coppery, but not purple in *C.enleensis*, Fig. 24); (2) femora black (middle of mesofemora and metafemora brown in *C.enleensis*); (3) elytral intervals convex basally, flat apically (interval convex throughout in *C.enleensis*); (4) apical lamella wider at base (narrower in *C.enleensis*, Figs 32A, C, D, 33A, C, D)

#### Description.

BL = 21.3–24.4 mm, BW = 8.0–8.7 mm. PL = 4.5–5.0 mm, PW = 5.0–5.5 mm, MW = 2.0–2.1 mm, ML = 1.7–1.9 mm. Head, elytra, venter, and legs dark and black; pronotum fully purple or greenish purple; antennae, labial and maxillary palpi, apex of mouthparts and tarsomeres dark brown.

***Head*.** Vertex finely punctate, pubescent, without a distinct glabrous area; antennae long, reaching middle of elytra; antennomere 3 ~ 1.5× longer than antennomere 4.

***Pronotum*** cordiform, PW/PL = 1.09–1.11 (Fig. 23), widest at apical third; anterior margin slightly concave, PAW/PBW = 0.83–0.93; lateral margins distinctly narrowed from middle to base, distinctly sinuate before posterior angles; anterior angles rounded, slightly projected forward; posterior angles nearly right angled, rounded at tips; disc gently convex, sparsely punctate and pubescent, with dense shallow transverse rugosities, without glabrous area in the middle; median line distinct, fine, not reaching anterior margin and base; basal foveae deeply arcuate, punctate and pubescent.

***Elytra*** elongate, EL/BW = 1.61–1.75, gently convex near anterior third, widest near posterior third, rounded at apex in males, subtruncate in females; striae with deep punctures; parascutellar striae well developed; parascutellar pores present; intervals convex at base, flat from middle to apex, densely punctate and pubescent; sutural angles obtuse and right; hind wings reduced.

***Venter*** densely punctate, pubescent, metepisterna (Fig. 15) short, MW/ML = 1.11–1.15; abdominal sternites III–VI with one setiferous puncture each side, sternite VII with one pair of setiferous punctures in males, two pairs in females; all abdominal sternites with distinct impressions laterally.

***Legs*** long and slender; tarsi nearly smooth dorsally.

***Male genitalia*.** Median lobe (Fig. 31B–E) long, strongly bent to ventral side; apical orifice opened dorsally, long and wide, not reaching basal bulb; in dorsal view, apical lamella triangular (Fig. 31A), distinctly bent to right side, longer than basal width, each side of middle apical lamella with a denticulation or absent on right side; in left lateral view, apical portion slightly bent dorsally at apex, basal orifice ~ 90° relative to preapical shaft; left paramere larger than right paramere, both helically curved; endophallus with flagellum thick and straight; basal part of flagellum with irregular bursa; apical part of flagellum with drop–shaped sclerite.

***Female genitalia*.** Bursa copulatrix (Fig. 39A–C) round, base with a distinct long digitiform protrusion; villous canal long, tortuously contorted, adhered to common oviduct; spermatheca and spermathecal gland absent.

#### Distribution.

(Fig. 40) China (Guangdong; Guangxi).

#### Remarks.

Chlaenius (Sphodromimus) wrasei was originally described as a member of the genus *Vachinius*. Azadbakhsh and Kirschenhofer (2019) moved it to the genus *Chlaenius*. However, in a result of this treatment, it became a junior homonym of Chlaenius (Lithochlaenius) wrasei (Kirschenhofer, 1997). Herein, we propose a new replacement name for the former – Chlaenius (Sphodromimus) davidi nom. nov., based on the first name of the well-known specialist on the ground beetles, David W. Wrase.

This species was described based on a single female from Datianding, Xinyi, Guangdong, China. Recently, one male and six females were collected from the type locality, and those specimens fit well with the original description and illustration of *C.davidi* nom. nov. Two more males were collected in Guangxi, which are identical to the holotype.

### ﻿ Chlaenius (Sphodromimus) flavofemoratus

Taxon classificationAnimaliaColeopteraCarabidae

Laporte, 1834

FCC2DE3B-0936-587D-A515-BAE3CFA8A826

Figs 7A–C , 14 , 22 , 30A–E , 38A–C , 40

Chlaenius flavofemoratus Laporte, 1834: 81; Chaudoir 1856: 244 (synonymized with Chlaeniusfemoratus Dejean, 1826); Chaudoir 1876: 93 (mention); Andrewes 1941: 308 (key to species; distinguished from Chlaeniusfemoratus Dejean, 1826); Andrewes 1947: 6 (Burma, Indo–China, The Malay Island, Hong Kong); Mandl 1992: 99 (Macrochlaenites; Java, Burma); Lorenz 1998: 318 (catalogue); Lorenz 2005: 338 (catalogue); Kirschenhofer 2017: 491 (catalogue); Azadbakhsh and Kirschenhofer 2019: 1 (transferred to subgenus Sphodromimus from subgenus Haplochlaenius)
nigricoxis Motschulsky, 1865: 339 (type locality: Hong Kong); Chaudoir 1876: 94 (redescription); Bates 1892: 312 (Bhamò, Palon, Karin Chebà, Laos, Java); Mandl 1992: 99 (synonymized with C.flavofemoratus Laporte,1834); Lorenz 1998: 318 (catalogue); Lorenz 2005: 338 (catalogue). Synonym.

#### Type locality.

Indonesia, Java.

#### Material examined.

Total 83 specimens. China – **Fujian**: 1 ♀ (IZAS), Fujian, Nanping, 1985.VI.7; 1 ♀ (IZAS), Fujian, Nanjing, 1991.V.15; **Guangdong**: 3 ♂♂ (IZAS), Guangdong, Haifeng, Jimingsi, 23.037605°N, 115.25343°E, 178.09m/2017.V.22, Y.Z. Liu, S.P. Yu leg., Inst. of Zoology; **Guangxi**: 1 ♂ (IZAS), Guangxi, Longsheng, Liluo, 1985. IV Jun Li leg.; 1 ♂ (IZAS), Guangxi, Guilin, Longsheng, 2003.VII.7 Jianxin Cui leg.; 1 ♀ (IZAS), Guangxi, Nanning, 1980.IV.21, Rongquan Cai leg.; 1 ♂ (IZAS), Guangxi, Jingxi, 840 m, 1998.IV.1 Chunsheng Wu leg.; **Guizhou**: 1 ♂ and 1 ♀ (IZAS), Guizhou, Luodian, 420 m, 1979.IV.16, Qingqiang Li leg.; 1 ♀ (IZAS), Guizhou, Luodian, 1979.V.1; **Hainan**: 1 ♂ (IZAS), Hainan, Baisha, Yinggeling, 2011.V.1, 600 m, light trap, Wenxin Lin leg., Inst. of Zoology; **Yunnan**: 4 ♂♂ and 1 ♀ (IZAS), Yunnan, Jinghong, Virgin Forest Park, Peacock Villa, 22.0304°N, 100.8763°E, 682 m/2021.VIII.6 N, along road Pingzhou Zhu leg., Inst. of Zoology; 1 ♂ (IZAS), Yunnan, Xishuangbanna, Botanical Garden, Chao Wu leg.; 1 ♀ (IZAS), Yunnan, Hekou, Qiaotou, Bajiaotian village/ 22.85348°N, 104.14211°E, 886 m, 2021.IV.21 N, Y. Xu, Z.Q. Yan coll., Inst. of Zoology; 1 ♂ (CAS), China, Yunnan Province, Tengchong, Shangying, Longwenqiao, field, beach, 25°01'19.9"N, 98°40'40.4"E/1290 m, 2003.X.20 D., H.B. Liang, X.C. Shi Coll., Institute of Zool., CAS & California Acad. Sciences; 1 ♂ and 3 ♀♀ (IZAS), Yunnan, Xishuangbanna, Menglun, Botanical Garden, 549 m, 21°56.035'N, 101°15.154'E /2005.V.25, Guo Zheng leg, Inst. of Zoology, CAS; 4 ♂♂ and 6 ♀♀ (CAS), Yunnan, Xishuangbanna, Menglun, Botanical Garden, 558 m, 21°55.035'N, 101°16.500'E /2007.V.20, Guo Zheng leg, Inst. of Zoology, CAS; 3 ♂♂ and 8 ♀♀ (IZAS), Yunnan, Xishuangbanna, Menglun, Botanical Garden, 558 m, 21°55.035'N, 101°16.500'E /2007.VII.10, Guo Zheng leg, Inst. of Zoology, CAS; 1 ♂ and 4 ♀♀ (CAS), Yunnan, Xishuangbanna, Menglun, Botanical Garden, 572 m, 21°54.646'N, 101°16.257'E /2007.I.10, Guo Zheng leg, Inst. of Zoology, CAS; 2 ♀♀ (IZAS), Yunnan, Xishuangbanna, Menglun, Botanical Garden, 2009.XII.1, Guo Tang leg, Inst. of Zoology; 2 ♀♀ (IZAS), China, Yunnan Prov., Nabanhe N.R. Guomenshan, alt.1150 m, 2009.V.6, Jiayao Hu, Ziwei Yin leg.; 1 ♂ (IZAS), Yunnan, Jinghong, Menghai, Nabanhe N.R. Guomenshan, Forest, 2009.VI.26, 1114 m/22.24644°N, 100.60610°E, pitfall, L.Z. Meng leg., Inst. of Zoology; 1 ♂ (IZAS), China, Yunnan, Hekou, Longpu, 240 m, 2011.IV.13D, 22.65404°N, 103.98193°E/Xinlei Huang leg., Inst. of Zoology, CAS; 1 ♂ (IZAS), Yunnan, Xishuangbanna, Menglun, Botanical Garden, 540 m, 21.92987°N, 101.24820°E, 2011.IV.21N/Hongbin Liang, Kaiqing Li leg., Institute of Zool., CAS; 1 ♂ (CAS), Yunnan, Xishuangbanna, Menglun, Botanical Garden, 540 m, 21.92987°N, 101.24820°E, 2011.IV.22/Yan Li leg., Institute of Zool., CAS; 1 ♂ (IZAS), Yunnan Prov. Menglun, Botanical Garden, vegetation, 21.91175°N, 101.28163°E /650 m, 2009.XI.15, Guo Tang Coll., Inst. of Zoology; 1 ♀ (IZAS), Yunnan, Xishuangbanna, Menglun, Botanical Garden, 2011.V.04, 560 m/ 21°55'39.66"N, 101°15'18.09"E, Jingxin Liu leg., Inst. of Zoology; 1 ♀ (IZAS), Yunnan, Xishuangbanna, 29 km NW, Jinghong, vic. Da Nuo You/ 22°12.41'N, 100°38.29'E, 790 m, 2009.V.16 leg. L. Meng, rice follow; 1 ♀ (IZAS), Yunnan, Xishuangbanna, 20km NW, Jinghong, vic. Man Dian (NNNR)/22°07.80'N, 100°40.05'E, 740 m, 2008.V.13 leg. A. Weigei rubb. plant; 1 ♂ and 1 ♀ (CAS), Yunnan, Lushui, Pianma, Gangfang, Xuetang 26.12218°N, 98.57546°E/1625 m,2005.V.16N, D. Kavanaugh, D.Z. Dong leg., Inst. of Zoology, CAS; 1 ♂ (CAS), China, Yunnan, Mengla, Biodiversity Corridor, 660 m, 2011.IV.25D, 21.40482°N, 101.63035°E /Xinlei Huang leg., G213 1999 km, Inst. of Zoology, CAS; 1 ♂ (IZAS), Yunnan, Yangbi, Pingpo, 1422 m, 25°35'33"N, 100°2'56"E/2002.VI.27, Min Wu leg.; 1 ♀ (CAS), China, Yunnan Province, Tengchong, Qushi Town, Xiaojiangqiao, riverside, 25°14'22.2"N, 98°37'38.0"E/1445 m, 2003.X.21, night, H.B. Liang, X.C. Shi Coll., Institute of Zool., CAS & California Acad. Science; 1 ♀ (CAS), Yunnan, Gaoligongshan, Nujiang Prefecture, 1500 m, 26°07.3'N, 98°34.5'E, 1998.X.14, D.H. Kavanaugh leg.; 2 ♂♂ (IZAS), Yunnan, Xishuangbanna, Menglun Town NO.55, 2009.V.04, Hu Li leg.; 4 ♀♀ (IZAS), Yunnan, Xishuangbanna, Menglun, Botanical Garden, 2012.V.16, L.Z. Meng leg., Inst. of Zoology; 1 ♂ (IZAS), Yunnan, Xishuangbanna, Menglun, 1982.IV.26, Linyao Wang leg.; 1 ♂ (IZAS), Yunnan, 1980.VI.5, Fen Liu leg.; 1 ♀ (IZAS), Yunnan, Gaoligong, Cikai Town, Pulahe joint of Nujiang, 27.74843°N, 98.66498°E/1530 m 2004.X.23, night, D. Kavanaugh, D.Z. Dong leg. Inst. of Zoology, CAS; 1 ♂ (IZAS), Yunnan, Lancang, Mafang By pitfall traps, 22.57925°N, 99.99849°E/1723 m, 2004.VI.16, W.B. Gu coll, Inst. of Zoology, CAS; 1 ♀ (CAS), Yunnan, Lushui, Liuku, Gaoligong Shan, 25°51'20"N, 98°50'58"E/800 m, 2002.IX.19, H.B. Liang, night, Sino-American Exped., Inst. of Zoology, CAS; 1 ♂ (CAS), China, Yunnan, Gaoligongshan, Nujiang Prefecture, Gangfang, Sancha Lukou/26 07.3'N, 98 34.5'E, 1500 m, 1998.X.12, D.H. Kavanaugh collector.; **Myanmar**: 1 ♂ (NHML), Carin Cheba, 900–1100 m, L. Fea/Fry Coll., 1905.100./*Chlaeniusflavofemoratus* Cast. = *nigricoxis* Mots., comp with type, H.E. Andrewes det.; **Vietnam**: 1 ♀ (IZAS), Tonkin, Hoa Binh, leg: A. de Cooman.

**Figures 11–16.**
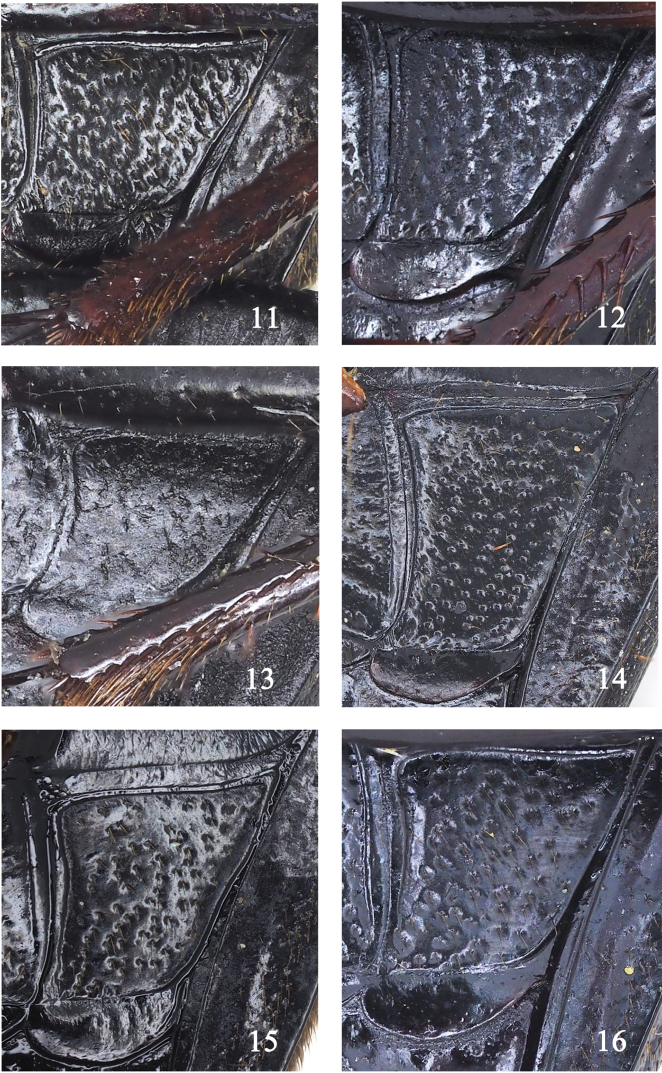
Metepisternum features of *Sphodromimus* spp. **11**Chlaenius (Sphodromimus) caperatus sp. nov., holotype **12**C. (S.) yinggelingensis sp. nov., holotype **13**C. (S.) pilosus (Casale, 1984), male **14**C. (S.) flavofemoratus Laporte, 1834, male **15**C. (S.) davidi nom. nov.; male **16**C. (S.) enleensis Mandl, 1992, male.

#### Diagnosis.

Pronotum metallic green to metallic coppery. PW/PL = 1.14–1.26; PAW/PBW = 0.76–0.92 (Fig. 22); pronotum subquadrate with anterior angles rounded, not projected forward; disc gently convex, sparsely punctate. Elytral intervals convex basally, flat apically, densely punctate and pubescent. Hind wings developed. Metepisterna long, MW/ML = 0.75–0.92 (Fig. 14). Distal half of femora red-brown, the rest of legs black. This species is similar to Chlaenius (Haplochlaenius) costiger Chaudoir, 1856, but intervals interval convex basally and flat apically, not costulate.

**Figures 17–24.**
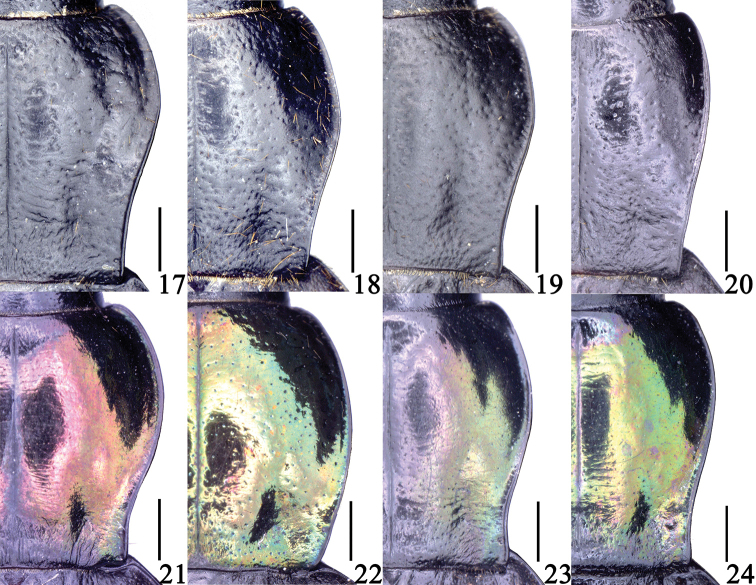
Pronotum features of *Sphodromimus* spp. **17**Chlaenius (Sphodromimus) caperatus sp. nov.; holotype **18**C. (S.) hunanus (Morvan, 1997) male **19**C. (S.) deuvei (Morvan, 1997); male **20**C. (S.) pilosus (Casale, 1984), male **21**C. (S.) yinggelingensis sp. nov.; holotype **22**C. (S.) flavofemoratus Laporte, 1834, male **23**C. (S.) davidi nom. nov., male **24**C. (S.) enleensis Mandl, 1992, male. Scale bars: 1.0 mm.

#### Description.

BL = 22.3–25.3 mm, BW = 8.5–10.4 mm, PAW = 3.5–4.0 mm, PBW = 4.3–4.6 mm, PW = 5.5–5.8 mm, PL = 4.5–4.9 mm, MW = 1.9–2.5 mm, ML = 2.4–3.0 mm. Head, elytra, and venter black; pronotum metallic green to metallic coppery; antennae, labial and maxillary palpi, apex of mouthparts and tarsomeres dark brown; distal half of femora red-brown, the rest of legs black.

***Head*.** Vertex finely punctate and pubescent, with a glabrous area in the middle; antennae long, reaching middle of elytra; antennomere 3 ~ 1.7× longer than antennomere 4.

***Pronotum*** subquadrate, PW/PL = 1.14–1.26 (Fig. 22), widest at apical four-ninth; anterior margin slightly concave, PAW/PBW = 0.76–0.92; lateral margins rounded or straight before posterior angles; anterior angles rounded, not projected forward; posterior angles obtuse; disc gently convex, sparsely punctate, without glabrous area in the middle; median line distinct, fine, reaching anterior margin and base; basal foveae deep, punctate and pubescent.

**Figures 25–27.**
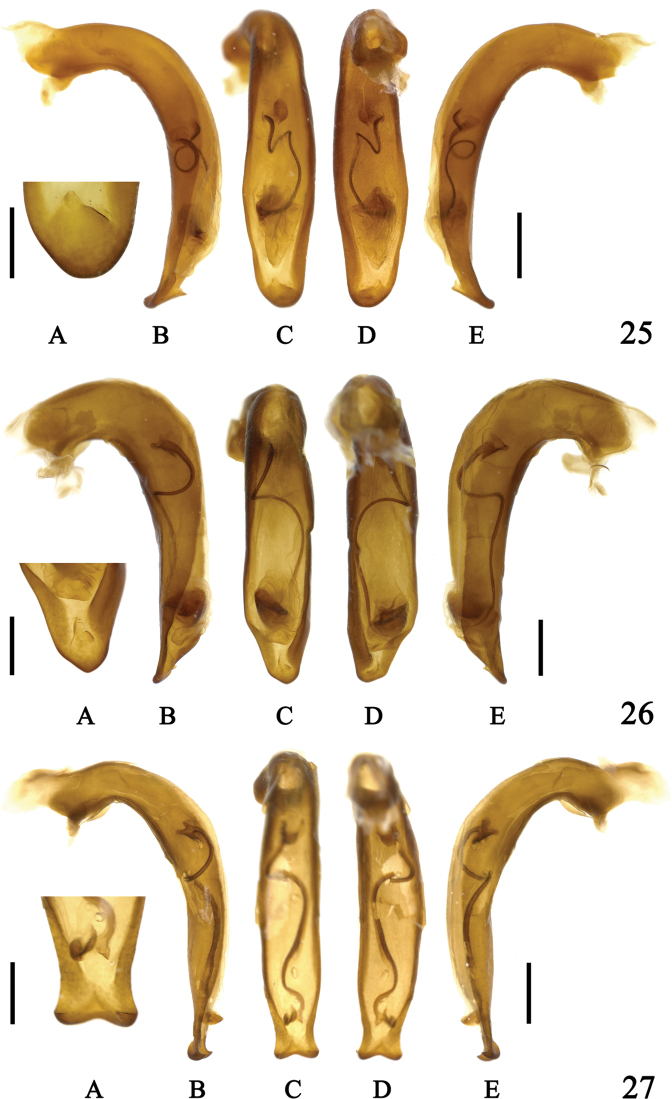
Aedeagus of *Sphodromimus* spp. **25**Chlaenius (Sphodromimus) caperatus sp. nov., holotype **26**C. (Sphodromimus) hunanus (Morvan, 1997) (Guangdong, Nanling) **27**C. (Sphodromimus) deuvei (Morvan, 1997) (Guangxi, Huaping) **A** apical lamella **B** left lateral view **C** dorsal view **D** ventral view **E** right lateral view. Left scale bars: 0.5 mm (**A**); right scale bars: 1.0 mm (**B–E**).

***Elytra*** elongate, EL/BW = 1.47–1.83; slightly convex, widest near posterior third, rounded at apex in males, subtruncate in females; parascutellar striae well developed; parascutellar pores present; striae with deep punctures; interval convex basally, flat apically, densely punctate and pubescent; sutural angles obtuse; hind wings developed.

***Venter*** densely punctate, pubescent; metepisterna (Fig. 14) long, MW/ML = 0.75–0.92; abdominal sternites III-VI with one setiferous puncture each side, sternite VII with one pair of setiferous punctures in males, two pairs in females.

***Legs*** long and slender; tarsi nearly smooth dorsally.

***Male genitalia*.** Median lobe (Fig. 30B–E) large, long, strongly bent to ventral side; apical orifice opened dorsally, long and wide, not reaching basal bulb; in dorsal view, apical lamella (Fig. 30A) slightly bent to left side, width slightly longer than length, apex rounded; in left lateral view, apical portion slightly bent ventrally at apex, left side near apical lamella with a large denticulation; internal sac with flagellum slender, apex with a helical sclerite, not reaching to apical orifice; left paramere larger than right paramere, both rounded; endophallus with flagellum fine and slightly bent; basal part of flagellum without a sclerite; apical part of flagellum with a drop–shaped bursa.

**Figures 28–30.**
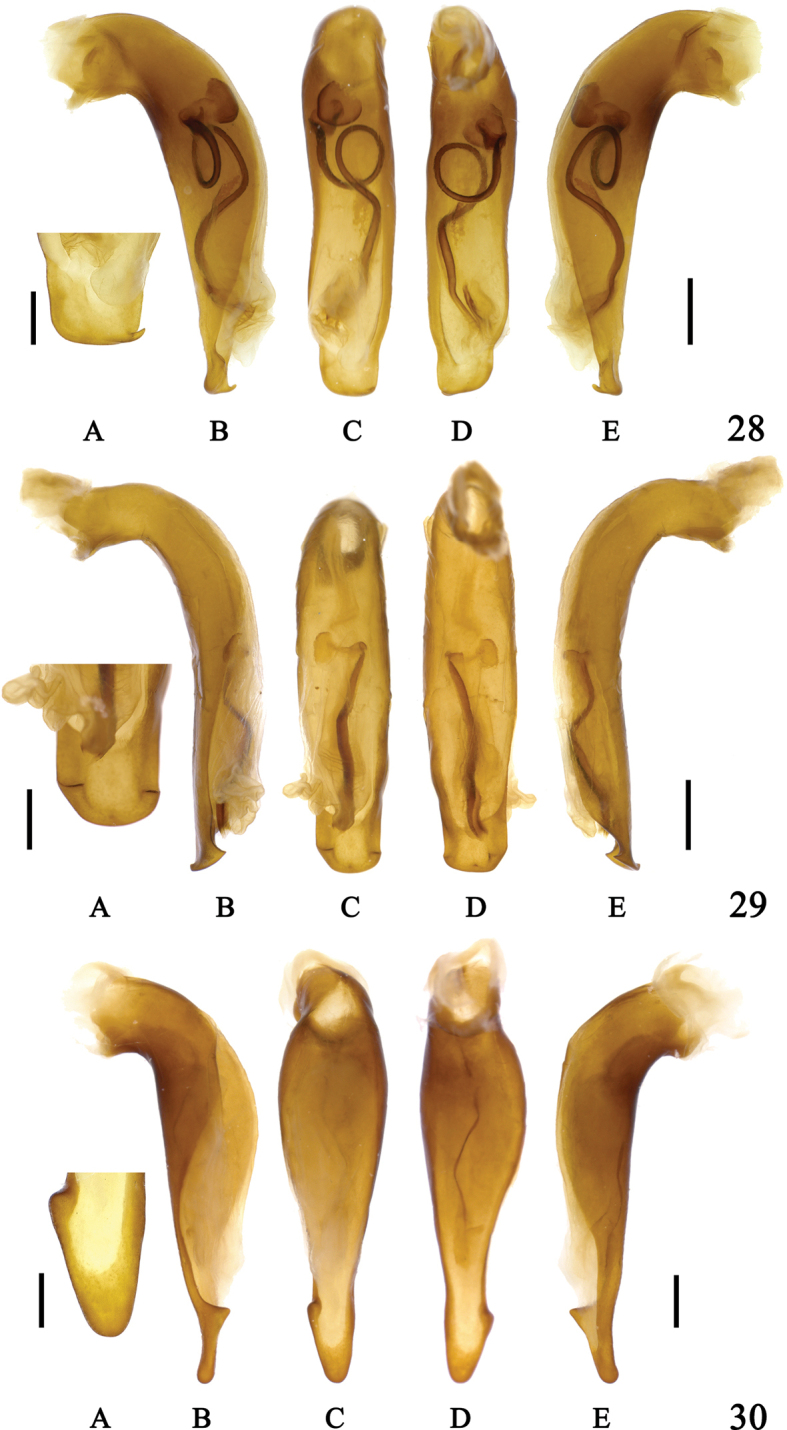
Aedeagus of *Sphodromimus* spp. **28**Chlaenius (Sphodromimus) pilosus (Casale, 1984) (Yunnan, Pingbian) **29**C. (Sphodromimus) yinggelingensis sp. nov, holotype **30**C. (Sphodromimus) flavofemoratus Laporte, 1834 (Yunnan, Menglun) **A** apical lamella **B** left lateral view **C** dorsal view **D** ventral view **E** right lateral view. Left scale bars: 0.5 mm (**A**); right scale bars: 1.0 mm (**B–E**).

***Female genitalia*.** Bursa copulatrix (Fig. 38A–C) very long, base with trapeziform protrusion; villous canal long, tortuously contorted, adhered to common oviduct; spermatheca and spermathecal gland absent.

#### Distribution.

(Fig. 40) China (Fujian; Guangdong; Guangxi; Guizhou; Hainan; Hong Kong; Yunnan), Indonesia, Laos, Myanmar, Vietnam.

#### Remarks.

Due to the fully developed hind wings and the shape of pronotum, this species is very special among species of *Sphodromimus*. It also has a wider distribution than other species. But the morphological characteristic of the apical lamella of the aedeagus, denticulate on the dorsal side, and the mentum with a bifid tooth show that the species belongs to *Sphodromimus*. The subspecies C. (Sphodromimus) flavofemoratus enleensis Mandl, 1992 was upgraded as a valid species (see below).

### ﻿ Chlaenius (Sphodromimus) enleensis

Taxon classificationAnimaliaColeopteraCarabidae

Mandl, 1992 stat. nov.

33485B29-28AD-5248-9B49-E1065845E20A

Figs 8 , 10A, B , 16 , 24 , 32A–F , 33A–E , 40

Chlaenius flavofemoratus enleensis Mandl, 1992: 100; Lorenz 1998: 318 (synonymized with C.flavofemoratus Laporte,1834, catalogue); Lorenz 2005: 338 (catalogue); Kirschenhofer 2017: 491 (as a subspecies of C.flavofemoratus; catalogue); Azadbakhsh and Kirschenhofer 2019: 1 (transferred to subgenus Sphodromimus from subgenus Haplochlaenius).
tamdaoensis Kirschenhofer, 2003: 32 (type locality: Vietnam, Tam Dao; genus Chlaenius, subgenus Haplochlaenius); Lorenz 2005: 342 (catalogue); Azadbakhsh and Kirschenhofer 2019: 1 (genus Chlaenius, subgenus Sphodromimus) syn. nov.

#### Type locality.

Indo Chine.

#### Material examined.

Total 5 specimens. **Vietnam**: ***Holotype***, Male (NHMB), Indo Chine coll. Dussault/ En-Le 1908/Chl (Macrochlaeniles) flavofemoratusssp.enleensis Dr. K. MANDL det. 1978/Holotype [red label]. ***Paratype*** (DWC, photo), 1 ♂ (IZAS), Vietnam, Tam Dao, 20–28.VI.1990, Dr. Blazicek lgt./Paratypus, Chlaenius (Haplochlaenius) tamdaoensis mihi det. Kirschenhofer 2001[red label]/COLL WRASE, BERLIN; 1 ♂ (IZAS), Vietnam, Tam Dao, 60 km NW Hanoi, 900 m, 1997 May-June, S. Ryabov [internal sac fully everted]; 1 ♂ (IZAS), Vietnam, Cao Bang, Nguen Binh, 800 m, 2003.V.13, S. Ryabov [internal sac partially everted]; 1 ♂ (IZAS), Tonkin, Hoa-Binh, leg. A. de Cooman [genitalia damaged by dermestes beetle].

#### Diagnosis.

Pronotum green to coppery. PW/PL = 1.06–1.12; PAW/PBW = 0.82–0.95 (Fig. 24); pronotum cordate with anterior angles rounded, not projected forward; disc sparsely punctate and pubescent, with shallow transverse rugosities. Elytral intervals gently convex throughout; densely punctate and pubescent. Hind wings reduced. Metepisterna short; MW/ML = 1.11–1.17 (Fig. 16). Apex of femora dark brown or yellow-brown, the rest of legs black.

#### Description.

BL = 21.7–24.1 mm, BW = 8.2–8.7 mm. PL = 4.5–4.9 mm, PW = 5.0–5.3 mm, MW = 2.0–2.1 mm, ML = 1.7–1.8 mm. Head, elytra, venter dark and black; pronotum green to coppery; antennae, labial and maxillary palpi, apex of mouthparts and tarsomeres dark brown; apex of femora dark brown or yellow-brown, the rest of legs black.

***Head*.** Vertex finely punctate, pubescent, without a distinct glabrous area; antennae long, reaching middle of elytra; antennomere 3 ~ 1.5× longer than antennomere 4.

***Pronotum*** cordiform, PW/PL = 1.06–1.12 (Fig. 24), widest at apical third; anterior margin slightly concave, PAW/PBW = 0.82–0.95; lateral margins distinctly narrowed from middle to base, slightly sinuate before posterior angles; anterior angles rounded, not projected forward; posterior angles nearly right angled, rounded at tips; disc gently convex, sparsely punctate and pubescent, with shallow, transverse rugosities, without glabrous area; median line distinct, fine, not reaching anterior margin and base; basal foveae deeply arcuate, punctate and pubescent.

***Elytra*** elongate, EL/BW = 1.58–2.22, gently convex near anterior third, widest near posterior third, rounded at apex in males; striae with deep punctures; parascutellar striae well developed; parascutellar pores present; intervals gently convex throughout, densely punctate and pubescent; sutural angles sharp at tips; hind wings reduced.

***Venter*** densely punctate, pubescent, metepisterna (Fig. 16) short, MW/ML = 1.11–1.17; abdominal sternites III–VI with one setiferous puncture each side, sternite VII with one pair of setiferous punctures in males; all abdominal sternites with distinct impressions laterally.

***Legs*** long and slender; tarsi nearly smooth dorsally.

***Male genitalia*.** Median lobe (Figs 32B–E, 33B–E) long, strongly bent to ventral side; apical orifice opened dorsally, long and wide, not reaching basal bulb; in dorsal view, apical lamella linear, longer than basal width (Figs 32A, 33A), distinctly bent to right side, with a denticulation laterally in the middle, left denticulation distinctly larger than the right one; in left lateral view, apical portion slightly bent dorsally at apex, basal orifice ~ 90° relative to preapical shaft; left paramere larger than right paramere, both helically curved; endophallus (Fig. 32F) with flagellum helically thick and straight; basal part of flagellum with irregular bursa; apical part of flagellum with triangular sclerite.

**Figures 31–33.**
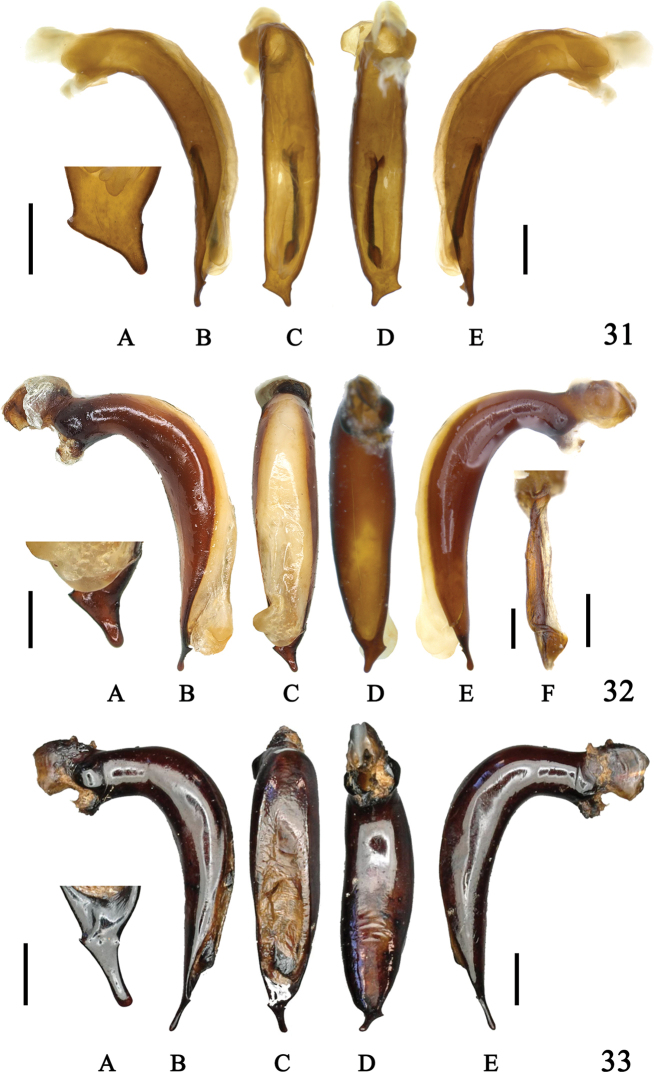
Aedeagus of *Sphodromimus* spp. **31**Chlaenius (Sphodromimus) davidi nom. nov. (Guangdong, Xinyi) **32**C. (Sphodromimus) enleensis Mandl, 1992 (Vietnam, Tam Dao) **33**C. (Sphodromimus) enleensis Mandl, 1992 (holotype, “Indo Chine”) **A** apical lamella **B** left lateral view **C** dorsal view **D** ventral view **E** right lateral view **F** endophallus. Left scale bars: 0.5 mm (**A**); right scale bars: 1.0 mm (**B–F**).

**Figures 34–36.**
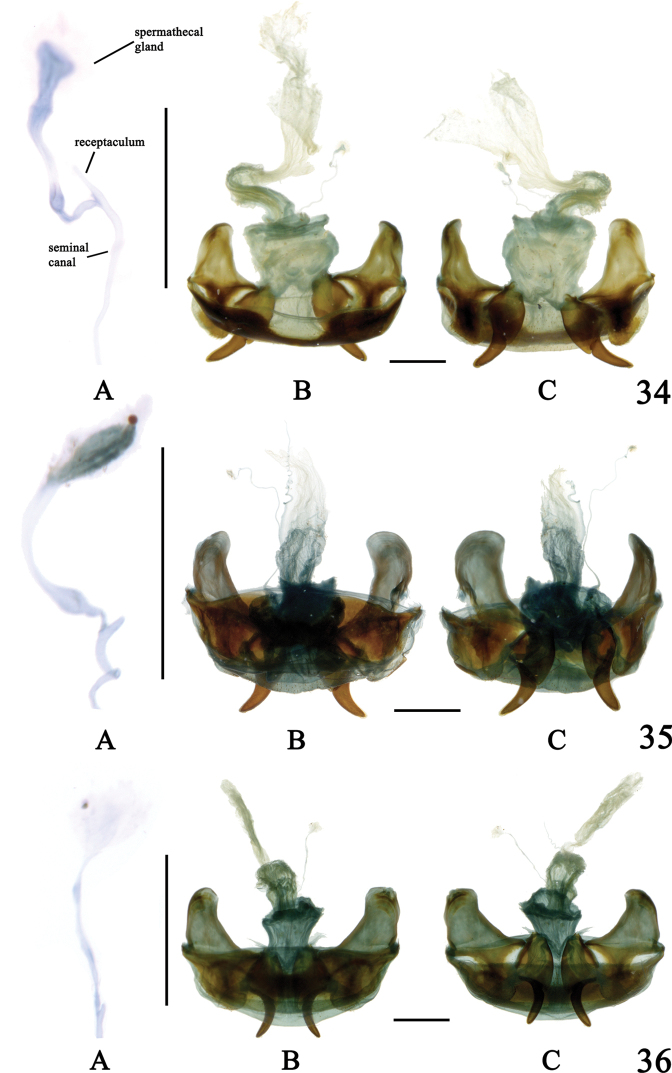
Internal reproductive system of females **34A–C**Chlaenius (Sphodromimus) caperatus sp. nov., paratype **35A–C**C. (Sphodromimus) hunanus (Morvan, 1997) **36A–C**C. (Sphodromimus) deuvei (Morvan, 1997) **A** spermatheca **B** dorsal view **C** ventral view. Vertical scale bars: 0.5 mm (**A)**; horizontal scale bars: 1.0 mm (**B, C**)

**Figures 37–39.**
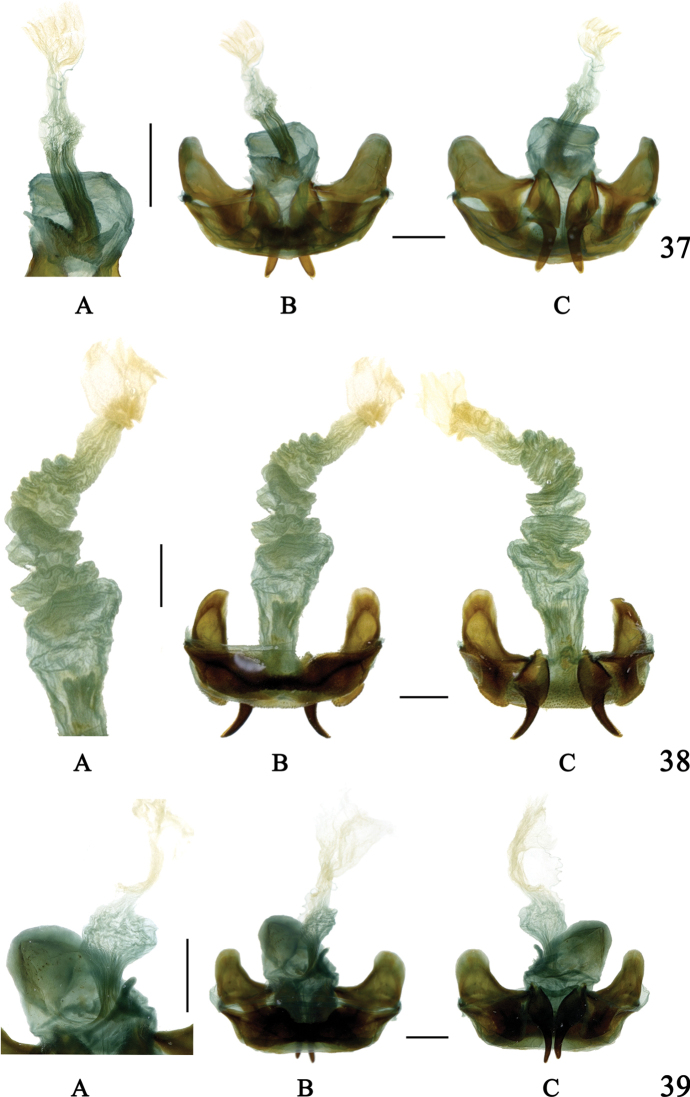
Internal reproductive system of females **37**Chlaenius (Sphodromimus) yinggelingensis sp. nov., paratype **38**C. (Sphodromimus) flavofemoratus Laporte, 1834 **39**C. (Sphodromimus) davidi nom. nov. **A** bursa copulatrix **B** dorsal view **C** ventral view. Scale bars: 1.0 mm.

***Female genitalia*** unknown.

#### Distribution.

**Figure 40.**
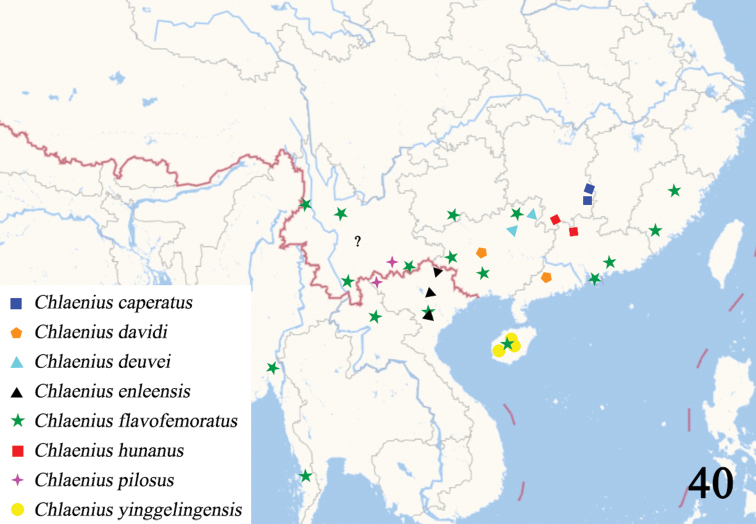
Distribution of Chlaenius (Sphodromimus) species in China and adjacent areas (a possible distribution of *C.enleensis*, En-le, Yunnan, represented by a question mark).

(Fig. 40) Vietnam (Indo Chine). We mark En-le, Yunnan on the map with a question mark ‘?’.

#### Remarks.

Lorenz (1998) and Lorenz (2005) proposed *C.flavofemoratusenleensis* Mandl, 1992 as a synonym of *C.flavofemoratus*, but Kirschenhofer (2017) treated it as a distinct subspecies. Based on its original description, its pronotum longer than *C.flavofemoratus*, and black femora indicated it probably represented a different species. After the examination of the holotype and its dissected genitalia, we find that it has the same apical lamella as *C.tamdaoensis*. As a consequence of this surprising discovery, the locality should also be critically revised. The labels of the holotype contain two localities (“En-le” and “Indo Chine”), the latter including today’s Vietnam. Based on the type locality of *C.tamdaoensis*, it is very unlikely that *C.flavofemoratusenleensis* also occurs in Yunnan’s En-le. Hence, we think that the label of En-le is likely to be the wrong one and may have been erroneously added, as it rarely happened in the historical collections of the NHMB (e.g., Caldara et al. 2022). We upgrade *C.enleensis* as a valid species and consequently treat *C.tamdaoensis* as synonym of *C.enleensis*.

## Supplementary Material

XML Treatment for Sphodromimus

XML Treatment for Chlaenius (Sphodromimus) caperatus

XML Treatment for Chlaenius (Sphodromimus) yinggelingensis

XML Treatment for Chlaenius (Sphodromimus) pilosus

XML Treatment for Chlaenius (Sphodromimus) davidi

XML Treatment for Chlaenius (Sphodromimus) flavofemoratus

XML Treatment for Chlaenius (Sphodromimus) enleensis
